# Sufficient Cav-1 levels in the endothelium are critical for the maintenance of the neurovascular unit in the retina

**DOI:** 10.1186/s10020-023-00749-9

**Published:** 2023-11-03

**Authors:** Yixin Wang, Mahmoud Halawa, Anupriya Chatterjee, Rachana Eshwaran, Yi Qiu, Yohanes Cakrapradipta Wibowo, Jianyuan Pan, Thomas Wieland, Yuxi Feng

**Affiliations:** 1 Experimental Pharmacology Mannheim, European Center for Angioscience (ECAS), Medical Faculty Mannheim, Heidelberg University, Ludolf-Krehl-Str. 13-17, 68167 Mannheim, Germany; 2 DZHK (German Centre for Cardiovascular Research), Partner Site Heidelberg/Mannheim, Mannheim, Germany

**Keywords:** Angiogenesis, Cav-1, Permeability, Retina, Vascular, Glial, Neuronal, VEGFR2

## Abstract

**Background:**

Caveolin-1 (Cav-1) is a pivotal protein in the plasma membrane. Studies on homozygous Cav-1 deficient mice revealed that Cav-1 is essential for endothelial function and angiogenesis in the retina. However, whether a reduction in Cav-1 content hampers the neurovascular unit (NVU) in the retina is unclear. Thus, this study examines the NVU in the retinas of heterozygous Cav-1 deficient (Cav-1^+/−^) mice and analyzes possible underlying mechanisms.

**Methods:**

The vascular, glial and neuronal components in the retina were evaluated using retinal morphometry, whole mount retinal immunofluorescence staining, histological analysis and optical coherence tomography. In addition, immunoblotting and immunofluorescence staining, subcellular fractionation, biotin labeling of cell surface proteins, and proximity ligation assay were employed to detect expression and localization of proteins in the retina or endothelial cells (ECs) upon knockdown of Cav-1 with Cav-1 siRNA.

**Results:**

Cav-1^+/−^ retinas showed a significant reduction in pericyte coverage along with an increase in acellular capillaries compared to controls at 8 months of age, but not at 1 month. A significant loss and obvious morphological abnormalities of smooth muscle cells were observed in 8-month-old Cav-1^+/−^ retinal arterioles. Macroglial and microglial cells were activated in the Cav-1^+/−^ retinas. A transient significant delay in retinal angiogenesis was detected in Cav-1^+/−^ retinas at p5, which was however no longer detectable at p10. The Cav-1^+/−^ retinas displayed increased vascular permeability and a notable reduction in VEGFR2 content at 8 months. In vitro, siRNA-mediated knockdown experiments in ECs revealed that the loss of Cav-1 in ECs resulted in decreased levels of VEGFR2, VE-Cadherin and their interaction at the plasma membrane as well.

**Conclusion:**

Our results indicate that a sufficient Cav-1 level over 50% of its normal abundance is vital for the proper localization of VEGFR2 and VE-cadherin, likely in a complex, at the plasma membrane, which is essential for the maintenance of normal NVU in the retina.

**Supplementary Information:**

The online version contains supplementary material available at 10.1186/s10020-023-00749-9.

## Introduction

Caveolins (Cavs), the key proteins embedded in the caveolae, are essential for protein internalization, lipid metabolism, and various cell signaling pathways (Cohen et al. 2004). Caveolae are small cave-like invaginated membrane structures that are present in most mammalian cell types and are particularly abundant at the surface of microvascular endothelial cells (ECs) (Enyong et al. 2022). They concentrate and compartmentalize numerous signal proteins and transport plasma proteins across the vascular endothelial barrier. The caveolin protein family includes three main subtypes: Cav-1, Cav-2, and Cav-3. Caveolin-1 has garnered the most attention due to its abundance in various cell types, especially in ECs (Minshall et al. 2003). Deficiency of Cav-1 in mice (Cav-1^−/−^) resulted in abnormal endocytosis, EC dysfunction, and increased systemic NO levels, leading to aberrant cardiopulmonary phenotypes (Razani et al. 2001; Zhao et al. 2002). Moreover, Miyawaki-Shimizu et al. found that the transient downregulation of Cav-1 in vivo by siRNA induced a loss of endothelial caveolae followed by an increase in vascular permeability in the lung (Miyawaki-Shimizu et al. 2006). Furthermore, homozygous Cav-1 deficiency has been linked to retinal diseases such as neuronal and vascular abnormalities (Sowa 2012; Schubert, et al. 2002). Cav-1^−/−^ mice showed a considerable increase in blood-retinal barrier (BRB) permeability around retinal venules, as well as reduced expression of pericyte marker NG2, indicating that homozygous loss of Cav-1 causes a BRB dysfunction (Gu et al. 2014a). In addition, Cav-1^−/−^ mice also show significant decreases in a- and b-wave amplitudes in electroretinography (ERG) (Li et al. 2012).

Angiogenesis, vascular maturation, and maintenance of the capillary network play a pivotal role in various physiological and pathological conditions (Carmeliet 2003, 2005). Numerous factors have been proven to regulate angiogenesis, and vascular endothelial growth factor (VEGF) is considered to be an imperative one (Gariano and Gardner 2005). The VEGF-VEGF receptor type 2 (VEGFR2) signaling cascade has been intensively studied in both health and disease (Apte et al. 2019). VEGFR2 is mostly found in vascular ECs, and loss of the VEGFR2 gene results in embryonic lethality due to poor development of hematopoietic and ECs (Shalaby et al. 1995). The VEGF-VEGFR2 signaling cascades are required for the maintenance of mature vessels (Baffert et al. 2004). VEGF is a key mediator of vascular hyperpermeability by binding and activating VEGFR2 and its downstream signaling components (Claesson-Welsh et al. 2021). Studies have shown that VEGFR2 is localized in the caveolae of the EC plasma membrane, and the knockdown of Cav-1 in ECs substantially reduces both basal and VEGF-induced VEGFR2 phosphorylation (Tahir et al. 2009; Feng et al. 1999). Labrecque et al. further reported that Cav-1 and VEGFR2 form a complex that undergoes rapid dissociation upon VEGF stimulation, thereby facilitating VEGFR2 phosphorylation, suggesting that Cav-1 serves as a negative regulator of VEGFR2 activity (Labrecque et al. 2003). Additionally, VE-Cadherin, a pivotal regulator of endothelial permeability, has been shown to interact with VEGFR2 within the caveolae. In the absence of Cav-1, this interaction is notably diminished under both physiological and pathological conditions (Lin et al. 2007). However, until now, it remains unclear whether a reduction in Cav-1 levels is already sufficient to hamper the VEGFR2 signaling and its interaction with VE-Cadherin for the maintenance of the function integrity of the neurovascular unit (NVU). Therefore, the present study focuses on the retinal NVU function in mice haplodeficient for the *CAV1* gene (Cav-1^+/−^). Interestingly, haplodeficient Cav-1^+/−^ mice exhibited similar abnormalities as Cav-1^−/−^ mice in the retina, which can be attributed to hampered VEGFR2 and VE-Cadherin functions in ECs already occurring at the reduced Cav-1 expression level.

## Materials and methods

### Antibodies and reagents

Primary antibodies used: lectin conjugated with fluorescein isothiocyanate (FITC) (L9381, Sigma-Aldrich, Taufkirchen, Germany); lectin conjugated with fluorescein isothiocyanate (TRITC) (L5264, Sigma-Aldrich, Taufkirchen, Germany); anti-Ionized calcium-binding adapter molecule1 (IBa1) (019-19741, Wako, Osaka, Japan); Anti-NG2 Chondroitin Sulfate Proteoglycan Antibody (AB5320, Sigma-Aldrich, Taufkirchen, Germany); monoclonal anti-actin, α-smooth muscle -Cy3™ antibody (α-SMA-Cy3) (C6198, Sigma-Aldrich, Taufkirchen, Germany); Rabbit-anti-VEGFR2 (55B11, Cell Signaling, Frankfurt, Germany), rabbit-anti-VE-Cadherin (D87F2, Cell signaling, Frankfurt, Germany), mouse-anti-caveolin-1 (sc-894, Santa Cruz Biotechnology, Heidelberg, Germany), rabbit-anti-albumin (55442, MP Bio, Eschwege, Germany), goat-anti-CD74 (sc-5438, Santa Cruz Biotechnology, Heidelberg, Germany), rabbit-anti-GFAP (Z0334, DAKO, Jena, Germany), mouse-anti-tubulin (T6557, Sigma-Aldrich, Taufkirchen, Germany), rabbit-anti-HSP90 (4874, Cell Signaling, Frankfurt, Germany), mouse-anti-Na/K-ATPase (MA3-915, ABR, Limerick, USA); goat-anti-human VEGFR2 (AF357, R&D, Nordenstadt, Germany); rabbit-anti-human VE-Cadherin (D87F2) XP® (2500, Cell Signaling, Frankfurt, Germany); mouse-anti-p120-catenin (sc-23872, Santa Cruz Biotechnology, Heidelberg, Germany). Secondary antibodies used: polyclonal swine anti-rabbit immunoglobulins/ FITC (F0205, Dako, Jena, Germany); polyclonal swine anti-rabbit immunoglobulins/ TRITC (R0156, Dako, Jena, Germany); rabbit-anti-mouse-HRP (Sigma-Aldrich, Taufkirchen, Germany) and goat-anti-rabbit-HRP (Sigma-Aldrich, Taufkirchen, Germany), and rabbit-anti-goat-HRP (Sigma-Aldrich, Taufkirchen, Germany); goat-anti-mouse Alexa Fluor™ Plus 555 (A32727, Invitrogen, Karlsruhe, Germany); goat-anti-mouse Alexa Fluor™ 488 (A21121, Invitrogen, Karlsruhe, Germany). Reagents: mounting medium (Roth, Karlsruhe, Germany); dispase (4942078001, Sigma-Aldrich, Taufkirchen, Germany); FCS (C-37350, Promocell, Heidelberg, Germany); endothelial cell basal medium (ECBM, C-22210, PromoCell, Heidelberg, Germany); OptiPRO SFM (serum-free medium) (2026928, ThermoFisher Scientific, Karlsruhe, Germany); lipofectamine RNAiMAX (2463615, Invitrogen, Karlsruhe, Germany); scramble siRNA (1027281, Qiagen, Hilden, Germany); Cav-1-specific siRNA 5` ACC ACG AAA CGT GAA GTT CAA 3´ (SI00299635, Qiagen, Hilden, Germany); collagenase I (Worthington, USA); protease inhibitor (11697498001, Roche, Germany); fractionation kit (78840, ThermoFisher Scientific, Darmstadt, Germany); Roti-block (Carl Roth, Karlsruhe, Germany); ECL detection reagent (ThermoFisher Scientific, Darmstadt, Germany); Sulfo-NHS-LC-biotin (ab145611, Abcam, Cambridge, UK); Neutravidin Agarose Resin beads (29200, ThermoFisher Scientific, Darmstadt, Germany); DAPI (D9542, Sigma-Aldrich, Taufkirchen, Germany).

### Animal care and handling

Animal care and experiments were in accordance with institutional guidelines and in compliance with the Association for Research in Vision and Ophthalmology statement and approved at the Medical Faculty Mannheim, Heidelberg University, Germany. Homozygous caveolin deficient (Cav-1^−/−^) and heterozygous Caveolin deficient (Cav-1^+/−^) mice on a C57Bl6 background were used for this study (Drab et al. 2001). Wild-type mice (Cav-1^+/+^) served as controls. The Genotype of the mice was determined using PCR techniques. Primers used for genotyping the: WT1 Cav1-296: 5` TTT ACC GCT TGT TGT CTA CGA 3´, WT2 Cav1-513: 5`TAT CTC TTT CTG CGT GCT GA 3`, KO1 Cav1-1452: 5`TCC TCC TAT TGC GGT GTG T 3`, and KO2 Neo 347: 5`CCT GCG TGC AAT CCA TCT TGT TCA ATG 3`.

### Retinal digestion & morphometry

The eyes of 8- and 1-month-old mice were fixed in 4% formalin for 24 h, and retinas were isolated under the dissection microscope. The isolated retinas were kept in ddH_2_O at room temperature overnight. Next, the retinas were incubated in 3% trypsin dissolved in 0.2 M Tris–HCl buffer (pH 7.0) at 37 °C for 2.5 to 3 h. Retinas were subsequently carefully transferred onto glass microscope slides and washed with drops of ddH_2_O until all neuronal cells were detached and the retinal vasculature was clearly observable under the microscope. The samples were stained with Period-Acid-Schiff (PAS) after the retinal vasculature had dried on the slide. Afterward, images of slides were taken under 40 × magnification. The total number of pericytes was counted in 10 randomly selected fields from the middle section of the retina using an image analyzing system, (CUE-2; Olympus Opticals, Hamburg, Germany), and this number was then normalized to the relative capillary density (number of cells per mm^2^ of capillary area). Additionally, the cumulative acellular capillaries were counted in 10 randomly selected microscopic areas of 40 × magnification using an integration ocular with a grid of 100 squares (Eshwaran et al. 2021).

### Histological analysis

Eyes from 8-month-old mice were fixed in 4% formalin for 48 h and embedded in paraffin. Vertical slices of 4 µm from near the optic nerves were stained with PAS and hematoxylin. The thickness of the entire retina, the outer nuclear layer (ONL), inner nuclear layer (INL), and ganglion cell layer (GCL) was measured, and the number of cells in each layer was quantified in the central and peripheral retina using microscope with an analysis program (Leica DM RBE, Bensheim, Germany).

### Whole-mount retinal immunofluorescence

Eyes from 5- and 10-day-old mice were utilized to evaluate the vascular morphology. The eyes were fixed in 4% Paraformaldehyde (PFA) on ice for 1 h and then retinas were isolated. The retinas were washed with phosphate-buffered saline (PBS) and further permeabilized and blocked in 1% bovine serum albumin (BSA) with 0.5% Triton-X-100 at room temperature for 1 h. Then, the retinas were incubated with Lectin-FITC (1:50) overnight at 4 °C. On the following day, the retinas were washed with PBS and post-fixed in 4% PFA for 30 min at room temperature. Finally, the retinas were mounted on microscope slides with a mounting medium. Images were acquired using a confocal microscope (Leica, Wetzlar, Germany).

To analyze retinal microglial activation, the 8-month-old retinas were incubated with Lectin-TRITC (1:50) and anti-IBa1 antibody (1:100) overnight at 4 °C. On the second day, the retinas were washed with PBS and incubated with the secondary antibody swine anti-rabbit FITC (1:20) for 90 min at room temperature. Retinal microglial were quantified in both the superficial and deep capillary layers. The number of Iba1 positive cells was counted and standardized by retinal area. For the evaluation of pericyte coverage, 8-month-old retinas were incubated with Lectin-FITC (1:50) and anti-NG2 antibody (1:200) overnight at 4 °C, then on the second day with the secondary antibody swine anti-rabbit TRITC (1:20) for 90 min at room temperature. Number of pericytes was standardized by capillary areas. Ten images from each retina were quantified using ImageJ software. To analysis of arteriolar diameter and SMA expression, 8-month-old retinas were incubated with α-SMA-Cy3 antibody (1:200). Images from 5–6 arterioles taken at an 800 µm radius centralizing optic nerve head (ONH) were analyzed. The number of smooth muscle cells in 80 µm length of arterioles was quantified using ImageJ software. For GFAP quantification, 8-month-old retinas were incubated with anti-GFAP antibody (1:500) overnight at 4 °C, then with the swine anti-rabbit FITC antibody (1:20). Finally, the retinas were mounted on microscope slides with a mounting medium (Roth). Images were taken using a confocal microscope (Leica, Wetzlar, Germany). Z-stack images were randomly obtained in the fields between the borders of 1/2 and outer 1/3 of the retinas. 3D viewers were used to illustrate the GFAP staining in astrocytes and Müller cells in the retina. Astrocytes in the superficial layer were imaged concurrently. Number of astrocytes was counted and standardized by retinal area, and the lengths of GFAP positive endfeet of astrocytes and Müller cells were measured and compared between groups.

### Optical coherence tomography (OCT) imaging

Mice were anesthetized with isoflurane and placed in front of the scanning laser ophthalmoscope (SLO) device (RETImap, Roland Consult, Brandenburgan der Havel, Germany) with a DTL electrode placed at the cornea. Mouse eyes were fitted with a + 100 dpt contact lens (Roland Consult, Brandenburg, Germany). The OCT B-scan cross-sectional images were obtained. The total retinal thickness (TRT) was defined as the width from the retinal nerve fiber layer (RNFL) to the retinal pigment epithelium (RPE) layer. Eight measurements were made at the same distance (600–800 µm) from ONH and averaged (Ferguson et al. 2013; Dysli et al. 2015).

### Isolation and culture of ECs

The use of human umbilical vein endothelial cells (HUVECs) was approved by the local medical ethics committee (Medical Faculty Mannheim, Heidelberg University, Germany). HUVECs were isolated from the umbilical cords of healthy newborn babies with the mothers' informed consent of the mothers. HUVECs were obtained by digestion with 1 mg/ml dispase, and suspended in 10% FCS to stop the enzyme activity. Cell suspensions were seeded in ECBM with 2% FCS and grown on 1% gelatin-coated T25 culture flasks in a humidified incubator at 37 °C and 5% CO_2_. The culture medium was changed after 2 h, and cells were split after they reached confluence and seeded in 1% gelatin coated T75 culture flasks in ECBM with 10% FCS. Cells until passage 3 were used for experiments.

### Transfection with siRNA

HUVECs were seeded in 1% gelatin-coated 6 cm dishes and cultured in ECBM containing 10% FCS at 37 °C overnight to 70–80% confluence. The cells were transfected using OptiPRO SFM (serum-free medium) and lipofectamine RNAiMAX following the manufacturer’s protocol. Scramble siRNA served as a control, and Cav-1-specific siRNA 5` ACC ACG AAA CGT GAA GTT CAA 3´ was used to knockdown Cav-1. Four hours post-transfection, the medium was changed to ECBM in 10% FCS. After 48 h, proteins were harvested for immunoblotting.

### Isolation and culture of retinal Müller cells

Müller cells were extracted from retinas of Cav^+/+^ and Cav^+/−^ mice between postnatal days 8–12. The eyes were rapidly enucleated and transferred into Dulbecco’s modified eagle medium (DMEM) containing glutamine (200 mM) and penicillin/streptomycin, and stored overnight at room temperature in the dark. The next day, the intact eyes were incubated in DMEM containing 0.1% trypsin and 70 U/ml collagenase I at 37 °C for 60 min. Subsequently, the eyes were placed in a 6 cm dish containing DMEM with 10% FCS, and retinas were isolated and dissociated with a pre-sterilized Pasteur pipette into small aggregates and seeded onto 6 cm culture dishes coated with collagen (6–8 retinas/ dish). The cell culture medium was remained unchanged for 5–6 days and then replenished every 2–3 days. Cells were cultured in a humidified incubator at 37 °C and 5% CO_2_.

### Subcellular fractionation and immunoblotting

Immunoblotting was performed using proteins extracted from HUVECs or retinas in RIPA buffer (50 mM Tris–HCl, pH7.4, 150 mM NaCl, 1 mM dithiothreitol, 1% Triton X-100, 1% sodium deoxycholate) containing protease inhibitor. Subcellular fractionation of cells was performed using a subcellular protein fractionation kit for cultured cells.

HUVECs were detached from the culture flasks using trypsin digestion, and the cells were collected. Soluble fraction was obtained by using cytoplasmic extraction buffer. Subsequently, the membrane extraction buffer was added. After incubation at 4 °C for 10 min, the membrane extract was obtained by centrifugation at 3000 × *g* for 5 min. 4 × Laemmli buffer was added into the lysates. The samples were then heated for protein denaturation at 95 °C. The proteins were separated by SDS-PAGE and transferred onto nitrocellulose membranes. After blocking with Roti-block at room temperature for 1 h, membranes were incubated with the primary antibodies overnight at 4 °C. On the second day, the membranes were washed and incubated with the corresponding secondary antibodies at room temperature for 1 h, followed by detection with an ECL detection reagent. The following primary antibodies were used: rabbit-anti-VEGFR2 (1:1000), rabbit-anti-VE-Cadherin antibody (1:1000), mouse-anti-caveolin-1 (1:500), rabbit-anti-albumin (1:5000), goat-anti-CD74 (1:1000), rabbit-anti-GFAP (1:1000), mouse-anti-tubulin (1:5000), rabbit-anti-HSP90 (1:1000), mouse-anti-Na/K-ATPase (1:500). Secondary antibodies used were rabbit-anti-mouse (1:10,000) and goat-anti-rabbit (1:10,000), and rabbit-anti-goat (1:10,000).

### Immunofluorescence staining

HUVECs on gelatin-coated 6 cm dishes were transfected with Cav-1 siRNA as described above. Then, the cells were passaged onto round glass coverslips 24 h after transfection and growing overnight in ECBM. Afterwards, the cells were fixed with 4% PFA for 10 min, permeabilized with 0.1% Triton X- 100 for another 10 min, and blocked with 1% BSA for 1 h at room temperature. Subsequently, cells were incubated with primary antibodies overnight at 4 °C. After washing three times with PBS the next day, the cells were incubated with secondary antibodies for 1.5 h at room temperature and then counterstained with DAPI. The samples were covered with Roti FluorCare mounting medium and images were acquired by a confocal microscope. Primary antibodies used were rabbit-anti-VEGFR2 (1:200), rabbit-anti-VE-Cadherin (1:200) and mouse-anti-Cav-1 (1:50). Secondary antibodies used were goat-anti-mouse Alexa Fluor™ Plus 555 (1:200), polyclonal swine-anti-rabbit FITC (1:20). Staining intensity was quantified using Image J software. To measure pixel densities, a line was drawn across the plasma membrane and cytoplasm, avoiding local areas with strong signals near the nuclear membrane. The pixels located at the membranes were considered as membranous expression, and average pixel values within cells were calculated as intracellular expression.

### Cell surface biotinylation

Cell surface biotinylation was conducted according to the previous report (Tarradas et al. 2013). Briefly, 48 h after transfection, plasma membrane proteins of HUVECs on 6cm^2^ disks were biotinylated by labelling with 0.5 mg/ml Sulfo-NHS-LC-biotin (Abcam, ab145611, Cambridge, UK) in PBS for 60 min. Then, the cells were washed three times with PBS containing 50 mM Glycine, then lysed in a lysis buffer containing 50 mM Tris/HCl pH 7.4, 150 mM NaCl, 1 mM EDTA, 1% (w/v) Triton X-100 and protease inhibitor cocktails. Afterward, the lysates were centrifuged and small amounts of supernatants were stored for input evaluation. The remaining supernatants were subsequently incubated with Neutravidin Agarose Resin beads overnight at 4 °C. Next day, following washing steps, beads were resuspended in 1 × gel loading buffer, and heated at 70 °C for 10 min. Finally, beads were removed by centrifugation, and supernatants were collected for immunoblotting.

### Proximity ligation assay (PLA)

HUVECs were pre-transfected with control siRNA or Cav-1 siRNA in 6-well plates as mentioned above. 24 h post-transfection, the cells were transferred into µ-Slide 15 Well 3D (10,000 Cells/well, IBIDI, Martinsried, Germany). The cells were kept growing for additional 24 h until they reached confluency, and fixed with 4% PFA in HBSS. PLA was performed according to the manufacturer’s protocol. Briefly, fixed cells were blocked with blocking buffer for 1 h at 37 °C. Next, the cells were incubated with goat-anti-human VEGFR2 (1:100) and rabbit-anti-human VE-Cadherin (1:100) overnight at 4 °C. Then, the cells were washed with TBST (0.05% Tween) and incubated with Navebody 1 and Navebody 2 in Navebody Diluent for 60 min at 37 °C. After washing, the cells were incubated with sequential enzymes at 37 °C. To visualize the co-localization of PLA at the plasma membranes, the cells were co-stained with mouse-anti-p120-catenin (1:200) and goat-anti-mouse Alexa Fluor™ 488 (1:200) and DAPI (1:10,000) as described previously. Quantification of PLA spots was performed using ImageJ. PLA spots at the plasma membrane were counted as described in 40 cells per condition (Hayashi et al. 2013).

### Statistical analysis

Data are represented as mean ± SEM. Student’s t-test or Analysis of Variance (ANOVA) with Tukey post-test was performed using GraphPad Prism 8 (GraphPad Software, USA). *p* values < 0.05 were considered statistically significant.

## Results

### Haplodeficiency in the *CAV1* gene induces a breakdown of the NVU in the mature mouse retina

**Fig. 1:**
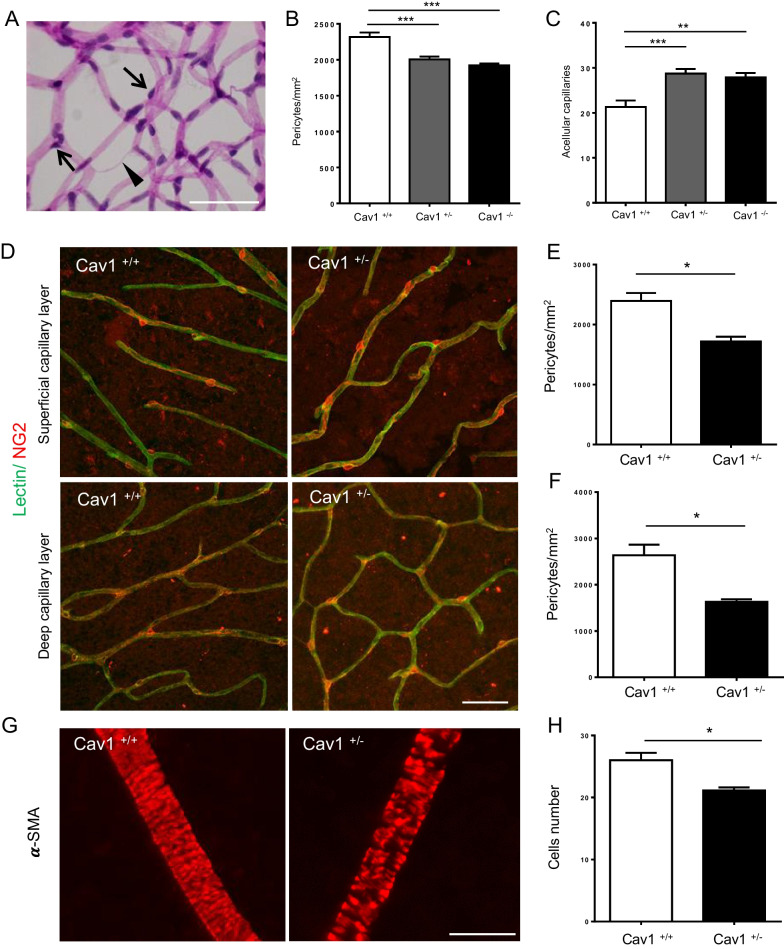
Haplodeficiency in the *CAV1* gene induces NVU breakdown in the mature mouse retina. Representative images of retinal digest preparations 8-month-old retinas (**A**). Arrows indicate pericytes and arrowhead shows an acellular capillary (AC). The quantification of pericyte coverage **B** and acellular capillary number **C** in 8-month-old retinas are demonstrated respectively. n = 7–8. Scale bar 50 µm. Representative whole-mount immunofluorescence images **D** show the pericyte coverage from Cav-1^+/+^ and Cav-1^+/−^ retinas in superficial **E** and deep **F** capillary layers. Red: NG2, pericyte marker; green: Lectin, vascular marker. n = 3. Scale bar 50 µm. Representative images of immunofluorescence staining of α-SMA in 8-month-old Cav-1^+/+^ and Cav-1^+/−^ retinas (**G**). Quantification of the number of smooth muscle cells on retinal arteriole (**H**). n = 3. Scale bar 50 µm. *p < 0.05, **p < 0.01, ***p < 0.001

To investigate whether insufficient Cav-1 affects the retinal vasculature in mature mouse retinas, we assessed retinal morphometry and quantified the pericyte coverage and segments of acellular capillaries (ACs) in mice at 8 months of age, a time point before mouse retinal vessels undergo degeneration (Feng et al. 2008) (Fig. 1). As expected, Cav-1^−/−^ retinas had significantly lower pericyte coverage compared with Cav-1^+/+^ retinas. Concomitantly, the absence of Cav-1 in Cav-1^−/−^ retinas significantly enhanced the formation of ACs compared with Cav-1^+/+^ retinas. Interestingly, Cav-1^+/−^ retinas showed similar alterations in pericyte coverage and AC formation as Cav-1^−/−^ retinas. Indeed, there were no obvious differences between Cav-1^−/−^ and Cav-1^+/−^ in terms of coverage of pericytes and formation of acellular capillaries (Fig. 1B, C). Retinal capillary density among Cav-1^+/+^, Cav-1^+/−^ and Cav-1^−/−^ showed no significant difference (Additional file 1: Fig. S1A). Additionally, we performed whole-mount retinal immunofluorescence staining with pericyte marker the NG2 to assess the pericyte coverage on retinal capillaries. As demonstrated with retinal morphometry, both the superficial and deep capillary layers showed lower pericyte coverage in Cav-1^+/−^ retinas compared to Cav-1^+/+^ retinas (Fig. 1D–F). The data suggest that retinal vessels are very susceptible to variation in Cav-1 content. A 50% depletion in Cav-1 can induce changes in retinal vascular morphometry similar to its complete absence, indicating that Cav-1 is an imperative molecule in the maintenance of normal retinal vasculature.

To determine whether the damage to the retinal vessels is initiated in mature vessels or is a consequence of malformations during vascular development in the retina, we analyzed pericyte coverage and AC formation in 1-month retinas, immediately after the retinal vessels are completely developed in mice. Given the analogous vascular phenotype in 8-month-old Cav-1^+/−^ and Cav-1^−/−^ retinas, our subsequent investigations were primarily centered on Cav-1^+/+^ and Cav-1^+/−^ retinas. Our analysis revealed no significant changes in pericyte coverage or AC formation between Cav-1^+/−^ and Cav-1^+/+^ retinas at 1 month (Additional file 1: Fig. S1B, C). No differences in capillary density and diameters of arterioles and venules could be observed between Cav-1^+/+^ and Cav-1^+/−^ retinas in both 1-month and 8-month-old mice on retinal digest preparations (Additional file 1: Fig. S1D–J). Abnormal retinal arterioles have been reported in homozygous Cav-1 deficient mice (Reagan et al. 2018). To determine whether heterozygous Cav-1 deficiency has a similar phenotype, the diameter of arterioles and coverage of smooth muscle cells on retinal arterioles labelled by α-smooth muscle actin (αSMA) were analyzed in 8-month-old Cav-1^+/+^ and Cav-1^+/−^ mice via whole-mount retinal immunofluorescence staining. The diameters of arterioles and venules in Cav-1^+/−^ retinas were comparable to controls (Additional file 1: Fig. SI, J). The diameters of arterioles and venules on retinal digest preparations (Additional file 1: Fig. S1E, F) were thicker than that in whole-mount retinal immunofluorescence staining (Additional file 1: Fig. S1I, J). As shown in Fig. 1G, smooth muscle cells on Cav-1^+/−^ arterioles were smaller, round and irregular, while the cells on Cav-1^+/+^ arterioles showed normal morphology, regular shape and distribution. Therefore, *CAV1* haplodeficiency resulted in a significant loss and obvious morphological alternation of smooth muscle cells on retinal arterioles (Fig. 1G, H).

The body weight and blood glucose of Cav-1^+/−^ mice were comparable to those Cav-1^+/+^ mice (Additional file 1: Fig. S1K–N), suggesting that there are no changes in obvious general metabolic parameters between these two groups. These results indicate that haplodeficiency in *CAV1* results in the aberrant maintenance of already established mature retinal vessels rather than a defect in retinal vascular development.

**Fig. 2:**
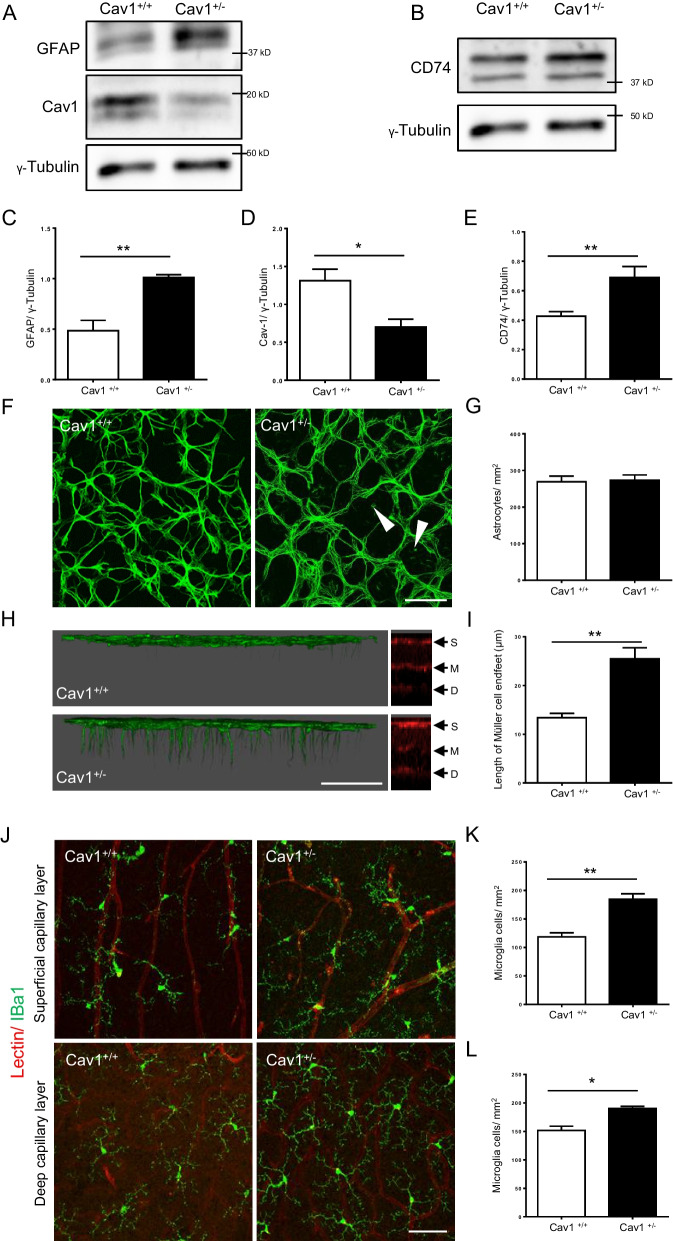
Haplodeficiency in the *CAV1* gene activates macroglial and microglial cells in the retina. The expression of GFAP (**A** and **C**), a marker of macroglial activation, CD74 (**B** and **E**), a marker of microglial activation, and Cav-1 (**A** and **D**) in the 8-month-old Cav-1^+/+^ and Cav-1^+/−^ retinas were analyzed by immunoblotting. γ-tubulin served as a loading control. Representative images (**A** and **B**) as well as quantifications obtained by pixel density assessments are shown. n = 6. Representative immunofluorescence images show the astrocyte coverage in Cav-1^+/+^ and Cav-1^+/−^ retinas and arrowheads show the endfeet of Müller cells (**F**). Quantification of the number of astrocytes coverage (**G**). n = 3. Scale bar 50 µm. **H** shows representative confocal z-stack topographical analysis images by immunofluorescence staining for GFAP in 8-month-old Cav-1^+/+^ and Cav-1^+/−^ retinas with Lectin staining showing S: superficial, M: middle and D: deep capillary layers. Quantification of the length of the GFAP positive cells penetrating into the deep capillary layer in retinas (**I**). n = 3. Scale bar 50 µm. Representative immunofluorescence images (**J**) show the microglial cell coverage from Cav-1^+/+^ and Cav-1^+/−^ retinas in the superficial (**K**) and deep capillary (**L**) layers. n = 3. Scale bar 50 µm. *p < 0.05, **p < 0.01

In the context of well-studied pathological conditions, such as diabetic retinopathy, the breakdown of the NVU triggers the activation of macroglial and microglial cells in the retina. Therefore, we further assessed the expression of GFAP, an activation marker of macroglia. GFAP was significantly upregulated by approximately twofold in the 8-month-old Cav-1^+/−^ retinas compared to controls (Fig. 2A and C). The results from immunofluorescence staining showed that there was no difference in the morphology and numbers of astrocytes between two groups (Fig. 2F and G). Astrocytes were regularly distributed in the superficial retinal layer in control group, whereas endfeet of Müller cells were evidently detectable between astrocytes (Fig. 2F). In control group, GFAP positive astrocyte endfeet were found only in the superficial capillary layer, similar to published data (Lin, et al. 2022). In contrast, Cav-1^+/−^ retinas showed intense GFAP staining in the cell endfeet, morphologically like Müller cells penetrating into the deep capillary layer, suggesting Müller cell activation (Fig. 2H, I).

CD74 was identified as a novel marker of activation of microglial cells (Feng et al. 2011). The expression of CD74 in the 8-month-old Cav-1^+/−^ retinas was found to be about twofold higher than that in the control Cav-1^+/+^ retinas (Fig. 2B and E). Due to the lack of appropriate CD74 antibodies for immunofluorescence staining in the mouse retinas, IBa1 was employed as a microglial marker to designate microglial in retinas (Wake et al. 2013; Andre et al. 2017). IBa1 positive microglia were found both in the superficial and deep capillary layers. An increase in IBa1 positive microglia in the superficial and deep capillary layers was detected in Cav-1^+/−^ retinas compared with controls (Fig. 2J–L). A reduction in Cav-1 expression in the Cav-1^+/−^ retinas was verified by immunoblotting (Fig. 2A and D. These results suggest that the activation of macro- and microglial cells is also associated with vascular abnormalities due to reduced Cav-1 expression. Together with the data described above, it implicates damage of the NVU in retinas in mice haplodeficient in the *CAV1* gene.

### Haplodeficiency in the *CAV1* gene enhances retinal thickness without triggering neurodegeneration

**Fig. 3:**
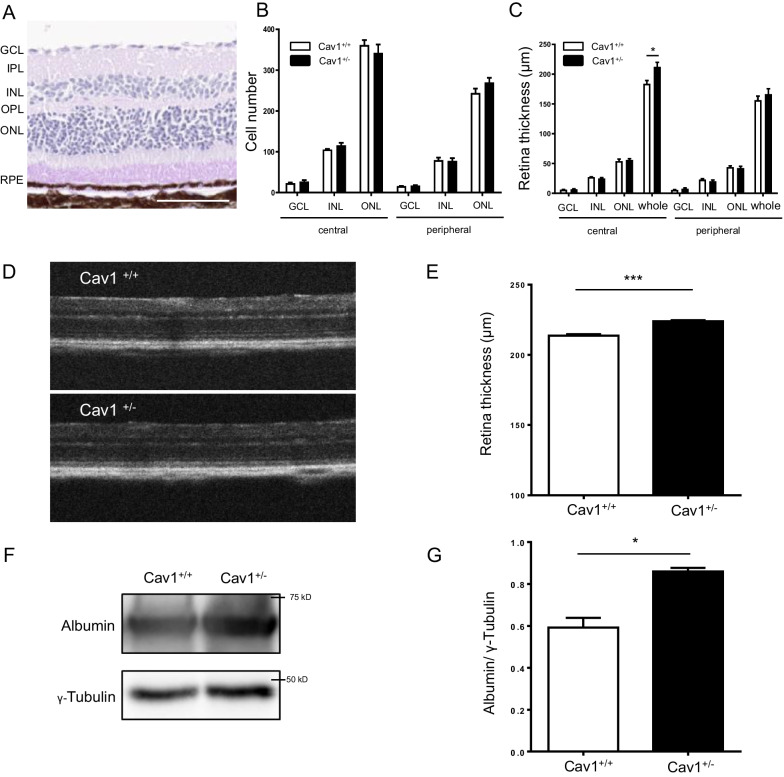
Haplodeficiency in the *CAV1* gene enhances retinal thickness without triggering neurodegeneration. Representative images of PAS staining of 8-month-old retina (**A**). Scale bar 100 µm. Quantification of the cell number **B** and the thickness of GCL, INL, ONL and whole retina in central and peripheral areas respectively **C** in Cav-1^+/+^ and Cav-1^+/−^ 8-month-old retinas. n = 5. Representative OCT images **D** and quantification **E** of whole retinal thickness of 8-month-old Cav-1^+/+^ and Cav-1^+/−^ retinas. n = 4–6. *GCL* ganglion cell layer, *IPL* inner plexiform layer, *INL* inner nuclear layer, *OPL* outer plexiform layer, *ONL* outer nuclear layer, *RPE* retinal pigment epithelium. Albumin content in the retina **F** and **G** was assessed by immunoblotting. n = 4. *p < 0.05, ***p < 0.001

After detecting vascular regression in 8-month-old Cav-1^+/−^ retinas, we further analyzed neuronal changes in these retinas by PAS staining (Fig. 3A), since neuronal and vascular damage are often coupled in the NVU in pathological conditions, such as diabetic retinopathy. As shown in Fig. 3B, there was no difference in cell counts between Cav-1^+/−^ and Cav-1^+/+^ mice in the ONL, INL and GCL, nor in central or peripheral sections of the retinas. Similarly, no differences in ONL, INL, or GCL thickness were found between Cav-1^+/−^ and Cav-1^+/+^ retinas in the central or peripheral areas (Fig. 3C). The thickness of whole central retinas, however, was significantly increased in Cav-1^+/−^ mice compared to Cav-1^+/+^ mice. In contrast, the whole peripheral retinal thickness in both groups was comparable. Further, retinal thickness was measured by OCT (Fig. 3D). The total retina thickness (TRT) in Cav-1^+/−^ mice was significantly higher than in controls, supporting the histological results (Fig. 3E). These data suggest that there is no obvious neuronal impairment in retinas of 8-month-old mice haplodeficient in the CAV1 gene. Nevertheless, the increased whole central retinal thickness might indicate an enhanced vascular permeability in Cav-1^+/−^ retinas.

As extravasation of albumin increases the entire retinal albumin content, we evaluated the levels of retinal albumin in 8-month-old Cav-1^+/+^ and Cav-1^+/−^ retinas by immunoblotting. We detected that the protein level of albumin in Cav-1^+/−^ mice was 45.4% higher than that in controls (Fig. 3F, G), validating the hypothesis that an increased retinal vascular permeability likely contributed to the increase in retina thickness in the Cav-1^+/−^ retinas.

### Reduced Cav-1 levels hamper early retinal vascular development

**Fig. 4:**
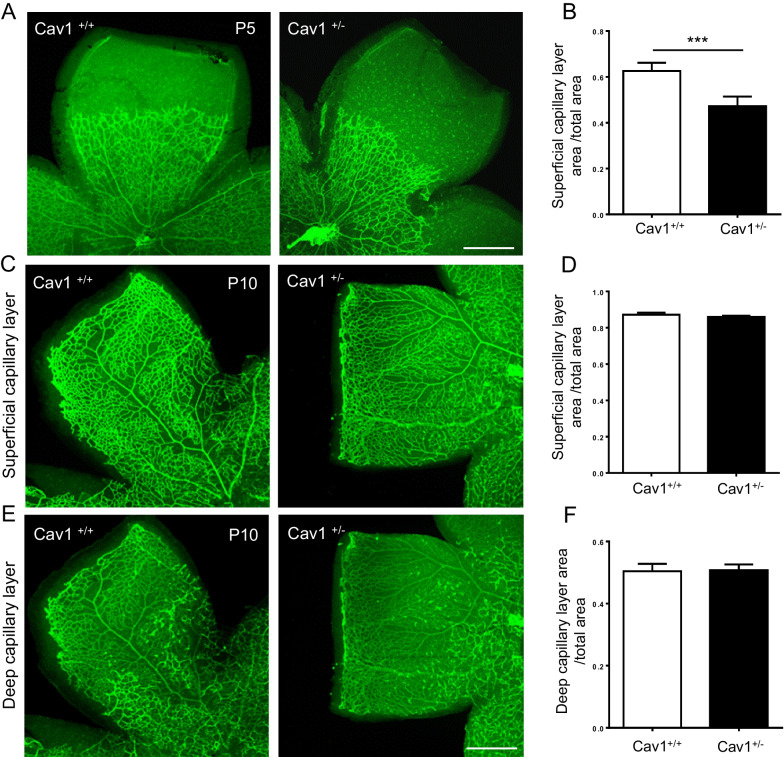
Reduced Cav-1 levels hamper early retinal vascular development. Retinal superficial capillary layer at postnatal day 5 (p5) (**A** and **B**, n = 7–10), superficial (**C** and **D**) and deep capillary (**E** and **F**) layers at p10 (n = 6) were assessed in retinal whole mounts stained with Lectin-FITC. Representative images are shown in **A**, **C** and **E**. Quantifications of the vascularized areas are presented in **B**, **D** and **F**. n = 6. Scale bar 400 µm. ***p < 0.001

We detected that all retinal vessels were completely developed in 1-month-old Cav-1^+/−^ mice compared to the Cav-1^+/+^ control mice. Nevertheless, a previous study showed that the ablation of Cav-1 (Cav-1^−/−^) was associated with severe retardation of retinal vascular development at postnatal day 5 (p5) (Gross et al. 2017). To study whether a 50% reduction of Cav-1 levels already evokes retardation of retinal vascular development, we firstly compared the superficial retinal vascular growth of Cav-1^+/−^ and Cav-1^+/+^ mice at p5. As shown in Fig. 4A, B Cav-1^+/+^ retinas demonstrated 62% outgrowth of the superficial capillary layer. In contrast, that of Cav-1^+/−^ retinas occupied only 47% of the entire retina, hence exhibiting a 25% retardation compared to the control retinas. The capillary density was slightly decreased in Cav-1^+/−^ retinas compared with Cav-1^+/+^ controls but did not reach statistical significance (Additional file 1: Fig. S2E). Furthermore, no differences in tip cell numbers, filopodia numbers and diameters of venules and arterioles were observed between Cav-1^+/−^ and control retinas (Additional file 1: Fig. S2C, D and S2F–H). To evaluate the effects of haplodeficiency in the *CAV1* gene on the development of the superficial and deep capillary layer, we further analyzed the retinal vasculature in mice at p10, which showed that the development of the superficial and deep layer capillary in Cav-1^+/−^ mice was comparable to controls (Fig. 4C–F). Additionally, to estimate whether the outgrowth of retinal vasculature was a result of differential development of the mice, we measured their body weight. The body weight of Cav-1^+/−^ mice did not differ from the controls either at p5 or at p10 (Additional file 1: Fig. S2A, B). Therefore, the data imply that the *CAV1* gene haplodeficiency transiently delays the retinal vascular outgrowth at early stages, and more than 50% of normal Cav-1 levels are required for regular early retinal vascular development.

### Vascular abnormalities induced by insufficient Cav-1 levels are likely mediated by aberrant VEGFR2 and VE-Cadherin localization

**Fig. 5:**
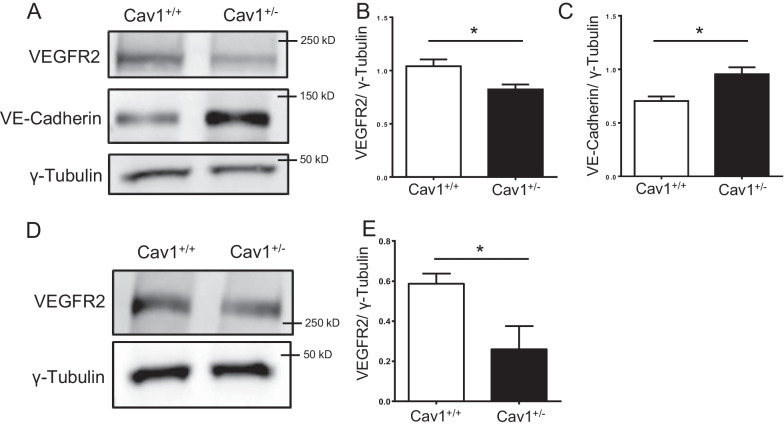
Insufficient Cav-1 levels alter the expression of VEGFR2 and VE-Cadherin in the retina. Expression of VEGFR2 and VE-Cadherin in 8-month-old Cav-1^+/+^ and Cav-1^+/−^ retinas (**A**–**C**) was assessed by immunoblotting (**A**–**C**). Additionally, VEGFR2 protein content was assessed in Müller cells isolated from Cav-1^+/+^ and Cav-1^+/−^ mouse retinas at p8-10 (**D** and **E**). γ-tubulin served as a loading control. Representative images (**A** and **D**) and quantifications obtained by pixel density assessments are shown (**B**, **C** and **E**). n = 4–6. *p < 0.05

VEGFR2 has been identified as a key mediator in vascular development and maintenance of mature vessels. Cav-1 and VEGFR2 are colocalized in caveolae, and Cav-1 knockdown reduced VEGFR2-mediated activation of downstream signaling in ECs (Gross et al. 2017). Based on the properties of VEGFR2, Cav-1, and their interaction, we evaluated the expression of VEGFR2 protein in Cav-1^+/−^ and Cav-1^+/+^ retinas of 8-month-old mice (Fig. 5A, B). The expression of VEGFR2 was significantly reduced by 25% in Cav-1^+/−^ retinas compared to controls, denoting that even a 50% reduction in the Cav-1 levels is sufficient to attenuate VEGFR2 protein expression in the retina. Besides ECs, VEGFR2 is also highly expressed in Müller cells in the retina correlated with non-caveolar caveolin-1 (Enyong et al. 2022; Saint-Geniez et al. 2008). The reduced expression of VEGFR2 in the retina may be caused by altered expression in Müller cells. Thus, we isolated Müller cells from Cav-1^+/−^ and Cav-1^+/+^ mouse retinas. Compared with Cav-1^+/+^ Müller cells, the expression of VEGFR2 was reduced by roughly 60% in Cav-1^+/−^ Müller cells (Fig. 5D, E).

Our data on the increased central retinal thickness and retinal albumin content indicate increased permeability in the Cav-1^+/−^ retina. An interacting partner of VEGFR2, VE-Cadherin contributes substantially to the regulation of endothelial tightness and thus vascular permeability. We therefore hypothesized that an aberrant VEGFR2 – VE-Cadherin signaling axis occurs in the Cav-1^+/−^ retina. Surprisingly, immunoblots showed a significant increase in VE-Cadherin expression in lysates of Cav-1^+/−^ retinas compared to WT controls (Fig. 5A and C).

**Fig. 6:**
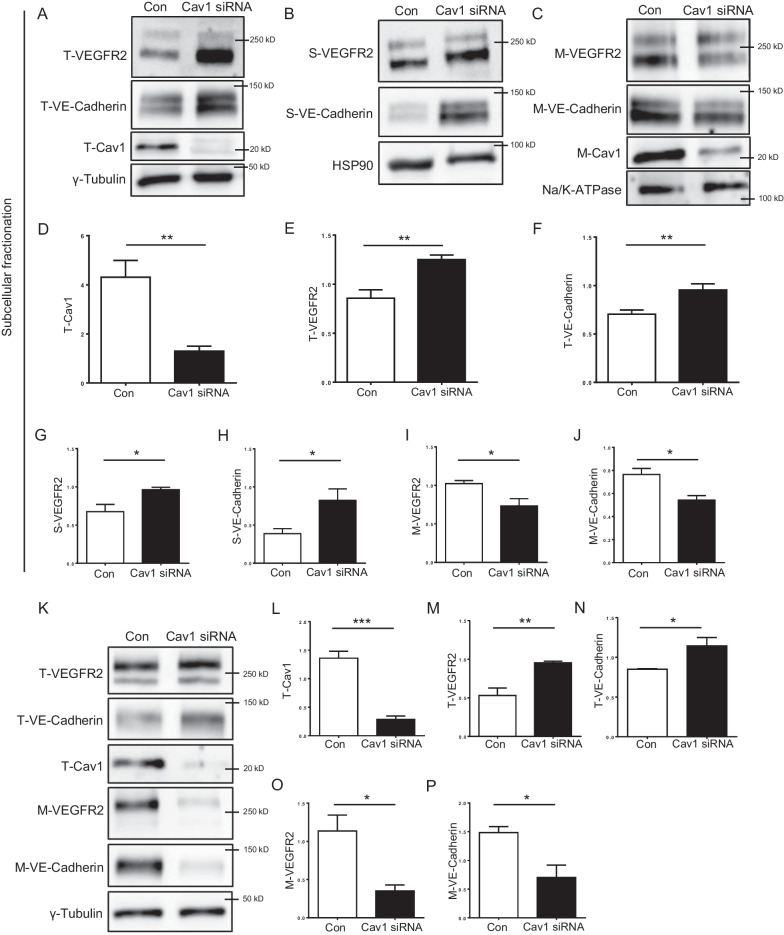
Vascular abnormality induced by insufficient Cav-1 is likely mediated by aberrant VEGFR2 and VE-Cadherin. Cav-1, VEGFR2 and VE-Cadherin protein content were assessed in total cell lysates (**A**, **D**–**F**), the soluble fraction **B**, **G** and **H** and the particular fraction **C**, **I** and **J** of Cav-1 depleted HUVECs and controls. Representative images (**A**–**C**), as well as quantifications obtained by pixel density assessments, are shown (**D**–**J**). Proteins from γ-tubulin, HSP90, and Na/K-ATPase served as loading control for total cell lysate, the soluble, and the membrane fraction, respectively. n = 4. Cav-1, VEGFR2 and VE-Cadherin were assessed in total cell lysates and in biotinylated cell surface proteins of Cav-1 depleted and control ECs (**K**–**P**). Representative images **K** and quantifications obtained by pixel density assessments are shown (**L**–**P**). n = 4. Con: control, Cav1 siRNA: Cav-1 depleted, T: total, S: soluble, M: membranous particular. *p < 0.05, **p < 0.01, ***p < 0.001

To address the mechanisms of hyperpermeability in retinal vessels exhibiting reduced VEGFR2 but increased VE-Cadherin expression in the retina, we further analyzed VEGFR2 and VE-Cadherin expression in HUVECs after siRNA-mediated Cav-1 knockdown using different membranous fractionation techniques. Figure 6A, D showed that siRNA transfection reduced Cav-1 protein levels by over 70% after 48 h. Interestingly, the total amount of VEGFR2 protein was increased in ECs after Cav-1 depletion, contrary to the result observed in the retinas (Fig. 5B). In comparison to control transfected cells, the expression of VEGFR2 was elevated by 45% in total cell protein lysates (Fig. 6A, E, K and M). In ECs, depletion of Cav-1 led to a 45% increase in the total VE-Cadherin. (Fig. 6A, F, K and N). We analyzed the subcellular distribution of VEGFR2 and VE-Cadherin using a subcellular fractionation kit, based on its relevance at the plasma membrane. After knocking down Cav-1, we observed an increase in the amount of VEGFR2 (~ 40%) as well as VE-Cadherin (~ 60%) in the soluble fractions (Fig. 6B, G and H). Interestingly, the opposite was observed in the membrane fraction of Cav-1-depleted ECs (Fig. 6C). A significant 25—30% decrease in VEGFR2 and VE-Cadherin protein levels was detected (Fig. 6I, J). Since the membrane fraction obtained from the kit consists of the plasma membrane and other intracellular membranes, we additionally isolated plasma membrane proteins using a cell-surface biotinylation method. The decrease in VEGFR2 and VE-Cadherin amounts (> 50%) was also detected in the fraction of the biotinylated surface proteins (Fig. 6K, O and P).

**Fig. 7:**
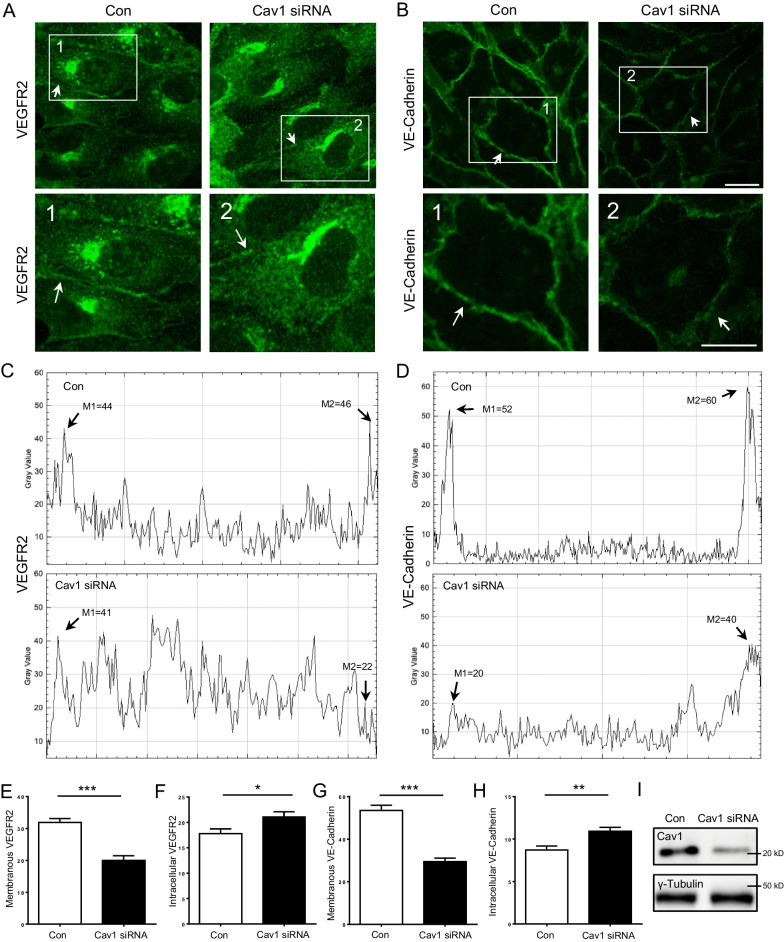
Depletion of Cav-1 decreases the endothelial expression of VEGFR2 and VE-Cadherin on the plasma membrane. Localization of VEGFR2 **A** and VE-Cadherin **B** by immunofluorescence staining in Cav-1 depleted ECs. Plasma membranes are indicated by white arrows and enlarged areas in the lower panels are labeled in the upper panels with white frames. M1 and M2 in pixel densities of line scans denote the plasma membranes for analyzing the expression of VEGFR2 **C** and VE-Cadherin (**D**). The quantification of plasma membranous and intracellular VEGFR2 **E** and **F** and VE-Cadherin **G** and **H** density are shown. **I** The content of Cav-1 in cell batches used for immunofluorescence staining was assessed by immunoblotting. n = 3. Scale bars 20 µm. *p < 0.05, **p < 0.01, *** < 0.001

To confirm the findings, we performed immunofluorescence staining of VEGFR2 and VE-Cadherin in ECs and quantified their distribution pattern, showing a significant decrease in plasma membrane levels of VEGFR2 and VE-Cadherin expression upon Cav-1 depletion. The data from immunofluorescence staining confirmed that the expression of VEGFR2 on the plasma membrane was significantly diminished whereas intracellular VEGFR2 levels were significantly enhanced in Cav-1 knockdown ECs (Fig. 7A, C, E and F). Similarly, the analysis of membranous and intracellular VE-Cadherin by immunofluorescence demonstrated that the presence of VE-Cadherin at the plasma membrane was significantly reduced whereas VE-Cadherin accumulated intracellularly in Cav-1 depleted ECs (Fig. 7B, D, G and H). The data hence suggest that sufficient Cav-1 levels are required for correct localization of both VEGFR2 and VE-Cadherin at the plasma membrane, possibly together in a complex.

**Fig. 8:**
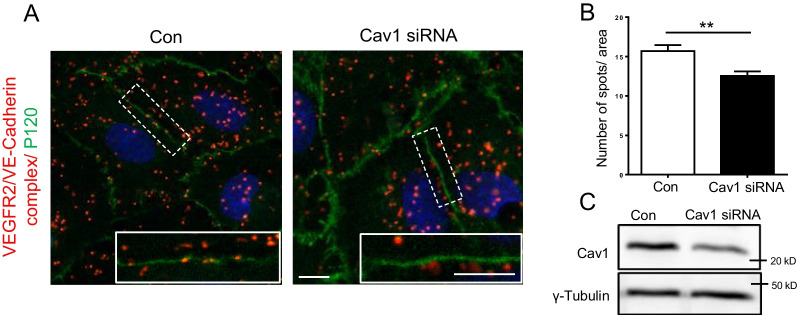
Cav-1 coordinates the interaction between VEGFR2 and VE-Cadherin at the plasma membrane**.** VEGFR2/VE-Cadherin PLA was performed in HUVECs transfected with control and Cav-1 siRNA. Immunostaining was performed using an anti-P120 antibody to visualize the plasma membrane (**A** and **B**). Representative images and high-magnification insets are shown to visualize the PLA products at the plasma membrane. Scale bar, 20 µm. **A** Quantification of the number of VEGFR2/VE-Cadherin complexes at the plasma membrane. **B** The data were obtained from three independent transfections. n = 40. The content of Cav-1 in cell batches used for the PLA was assessed by immunoblotting (**C**). **p < 0.01

To assess the complex between VEGFR2 and VE-Cadherin at the plasma membrane, we performed in situ PLA and stained the plasma membrane with an anti-p120 catenin antibody. Indeed, VEGFR2/VE-Cadherin complexes were detected at the plasma membrane in the control transfected cells. The number of these VEGFR2/VE-Cadherin complexes at the plasma membrane was however significantly decreased in Cav-1 depleted ECs (Fig. 8A, B). These findings further strengthen the notion that Cav-1 coordinates the interaction between VEGFR2 and VE-Cadherin and their placement in the plasma membrane.

## Discussion

In this study, we demonstrated for the first time that haplodeficiency in the *CAV1* gene in the mouse, 50% loss in retinal Cav-1, results in delayed postnatal retinal angiogenesis at p5 similar to Cav-1^−/−^ mice (Gross et al. 2017), which was however fully recovered at p10 in Cav-1^+/−^ mice. Furthermore, a breakdown of the NVU, including pericyte loss and increased segments of acellular capillaries, vascular hyperpermeability, and activation of macro- and microglial cells was observed in mature retinas at the age of 8 months. Surprisingly, these phenotypic changes in the Cav-1^+/−^ mouse in the retina resemble changes reported also from the homozygous Cav-1 deficient mice. These findings point out the relevance of a sufficient Cav-1 expression in the early stage of retinal vascular development. In the retina, Cav-1 is predominantly expressed in Müller cells and vascular cells, in particular in ECs (Li et al. 2012; Klaassen et al. 2009; Kim et al. 2006). Regarding postnatal vascular development, Cav-1 can be detected in the superficial retinal vessels and the hyaloid vessels already between p0 and p5. After p8, Cav-1 expression is markedly increased in the developing Müller glia in the retina (Gu et al. 2014b). Since Müller cells are not yet fully developed in the p5 retinas, our results are in agreement with a critical expression level of endothelial Cav-1 and the formation of endothelial caveolae in the vascular development up to p5 (Gross et al. 2017).

The observed alterations in the mature retina at 8 months of age are apparently not a consequence of consistent aberrant retinal development, since retinas at p10 showed normal vasculature. It is well known, that homozygous Cav-1 deficient mice exhibit severe defects in pathological endothelial permeability (Chang et al. 2009). There are, however, few studies assessing retinal vascular alterations in homozygous Cav-1 deficient mice (Gu et al. 2014a; Reagan et al. 2018). According to Gu et al., global complete Cav-1 ablation causes retinal venous expansion and decreased expression of pericyte marker NG2, but increased expression of another mural cell marker, αSMA, in venules. However, the coverage of pericytes in retinal capillaries was not assessed in Gu’s study, and the exact age of the mice used in the study was not mentioned. In our study, reduced coverage of pericytes was observed in retinas of 8-month-old but not 1-month-old Cav-1^+/−^ mice. Cav-1^+/−^ mice did not show alterations in venule and arteriolar diameters in either 8- or 1-month-old retinas. However, the coverage of smooth muscle cells decreased significantly in arterioles of 8-month-old Cav-1^+/−^mice, along with the morphological alteration. Our results from Cav-1^+/−^ mice are in line with the report by Reagan et al. who conducted studies on Cav-1^−/−^ mice, showing the essential role of sufficient Cav-1 levels in proper vascular function. We also observed increased permeability by detecting higher albumin levels in the Cav-1^+/−^ retinas compared with wild-type controls. The increased total retinal thickness in Cav-1^+/−^ retinas supports the notion of hyperpermeability. Hyperpermeability in Cav-1^+/−^ retinas in our study is in accordance with the study by Gu et al. showing increased BRB permeability in Cav-1^−/−^ mice (Gu et al. 2014a). Caveolin-1 localizes widely in vascular system including ECs and pericytes (Virgintino et al. 2002). Its function in pericytes has not been intensely investigated, and thus is still not elucidated. Whether the vascular dysfunction in Cav-1^+/−^ retinas is correlated with insufficient Cav-1 in pericytes, needs further investigations.

The activation of macro- and microglial cells in the 8-month-old Cav-1^+/−^ retinas further supports the finding of vascular damage. Such activation is common in pathological conditions such as diabetic retinopathy (Eshwaran et al. 2021; Wang et al. 2014). It remains unclear whether the activation is a result of the alterations in the endothelium or linked to non-caveolar Cav-1 deficiency. Vascular damage can for example occur as a response to neuronal degeneration in the retina (Rossino et al. 2019). Indeed, it has been reported that Cav-1^−/−^ mice exhibit abnormal retinal neuronal function as evaluated by ERG, showing decreased a-wave and b-wave amplitudes in comparison to the wild-type controls (Li et al. 2012). We therefore evaluated neuronal degeneration in the Cav-1^+/−^ retinas by counting neuronal cells in the GCL, INL, and ONL. The number of neuronal cells in the Cav-1^+/−^ retinas in our study did not differ from the wild-type retinas, indicating that vascular damage in Cav-1^+/−^ mice might not be a consequence of neuronal degeneration. Based on the observation that the angiogenesis in Cav-1^+/−^ retinas is hampered when Cav1 is not yet expressed in the Müller glia cells and that a loss in VE-Cadherin in endothelial adherence junctions is well known driving force for vascular damage and hyperpermeability in the retina (Lin et al. 2007; Davidson et al. 2000; Rangasamy et al. 2011; Smith, et al. 2020), we speculate that the activation of macro- and microglial cells follows endothelial damage rather than the reverse.

VEGF-induced activation of VEGFR2 leads to endothelial adherence junction remodeling via a signaling cascade involving specific phosphorylation of tyrosine residues on VEGFR2 (Y949) and VE-Cadherin (Y685) (Smith, et al. 2020). Functional VEGFR2 is localized in endothelial caveolae and directly interacts with Cav-1. The activation of VEGFR2 induces a rapid disassociation of Cav-1 and VEGFR2, leading to a clathrin-dependent internalization of VEGFR2 (Labrecque et al. 2003). Based on the crucial role of Cav-1 in endothelial vesicular trafficking, signal transduction, as well as its interaction with VEGFR2, we assessed the expression of VEGFR2 and its downstream effector VE-Cadherin in Cav-1^+/−^ retinas. While we detected a significant decrease in the protein amount of VEGFR2 in the retinas of 8-month-old Cav-1^+/−^ mice, the total amount of VE-Cadherin was significantly increased, which is, on the first view, in apparent contrast to the observed hyperpermeability. Our further analysis revealed that the loss of the retinal VEGFR2 in the 8-month-old Cav-1^+/−^ mice, likely originates from a decreased expression in Müller glia cells. In contrast, the increase of the total amount of VE-Cadherin originates from ECs due to its restricted and highly specific expression pattern (Vestweber 2008). To get a deeper mechanistic insight, we further analyzed Cav-1 depletion in cultured ECs using siRNA-mediated knockdown. In the ECs, the loss of Cav-1 was associated with an increase in the total protein amount of VEGFR2 and VE-Cadherin. Using several distinct fractionation as well as optical localization methods, we consistently detected increased VEGFR2 and VE-Cadherin protein amounts in intracellular compartments, whereas the amounts of VEGFR2 and VE-Cadherin present at the plasma membrane reduced by more than 50%. The higher amounts detected in intracellular compartments are likely feedback to the hampered Cav-1-dependent insertion of both proteins into the plasma membrane and the formation of caveolae. Taking the direct signaling from VEGFR2 to VE-Cadherin for adherence junction remodeling into account, the reduced VEGFR2 and VE-Cadherin levels at the cell membrane in Cav-1^+/−^ ECs likely cause the increased endothelial permeability followed by vascular damage and the breakdown of the NVU. This interpretation is further supported by data obtained in Cav-1^−/−^ mice, which exhibit a dramatically increased microvascular permeability, and defects in capillary adhesion to the basement membrane (Schubert, et al. 2002). Nevertheless, Müller cells are the main cell type responsible for the maintenance of water homeostasis in the retina and contribute to the development of vascular damage in pathological conditions (Bringmann et al. 2006; Reichenbach and Bringmann 2013). Thus, an open question remains, whether the loss of the VEGFR2 in Müller cells also contributes to the observed hyperpermeability.

## Conclusion

In conclusion, our findings provide evidence that sufficient amounts of endothelial Cav-1 are crucial for the maintenance of a proper NVU in the retina. A 50% loss, caused by genetic ablation of one *CAV1* allele, is sufficient to induce retinal vascular damage, including pericyte loss, formation of acellular capillaries, vascular hyperpermeability, and activation of macro- and microglial cells. Mechanistically, abnormal VEGFR2 signal transduction to VE-Cadherin and a reduction in adherence junction stability are likely the initial triggers to the pathological alterations occurring in the Cav-1^+/−^ retinas. The preservation of sufficient Cav-1 levels and its potential downstream signaling pathways may serve as a target in treating retinal vascular disorders.

### Supplementary Information

**Additional file 1: Figure S1.** Vasculature phenotype in 1-month-old retinas, body weight in 1- and 8-month-old Cav-1^+/−^ mice. The quantification of capillary density in 8-month-old Cav-1^+/+^, Cav-1^+/−^ and Cav-1^−/−^ retinas (**A**). n = 7–8. The quantification of pericyte coverage (**B**), acellular capillary number **C** and capillary density **D** in 1-month-old Cav-1^+/+^ and Cav-1^+/−^ retinas. n = 7–8. The quantification of arterioles **E** and **G** and venules **F** and **H** diameters in 8- **E** and **F** and 1-month-old **G** and **H** Cav-1^+/+^ and Cav-1^+/−^ on retinal digest preparation. n = 5. The quantification of arterioles **I** and venules **J** diameters in 8-month-old Cav-1^+/+^ and Cav-1^+/−^ in whole-mount immunofluorescence staining retinas. n = 3. Body weight (K and L) and blood glucose (M and N) of 8- (K and M) and 1-month-old (L and N) Cav-1^+/+^ and Cav-1^+/−^ mice. n = 8. **Figure S2. **Body weight and vascular sprouts of Cav-1^+/−^ retinas at p5. Quantification of the body weight of Cav-1^+/+^ and Cav-1^+/−^ mice at p5 (**A**) and p10 (**B**). n = 6. Quantifications of the retinal arteriolar **C** and venular **D** diameters and capillary density **E** at p5 retinas. n = 6. Representative images **F** and quantifications of retinal vascular tip cells **G** and filopodia **H** of Cav-1^+/+^ and Cav-1^+/−^ retinas at p5. n = 6. Scale bar 50 µm.

## Data Availability

The authors agree to make the data available upon request.

## Abbreviations

α-SMA: α-Smooth muscle actin
AC: Acellular capillary
BRB: Blood-retinal barrier
BSA: Bovine serum albumin
Cav-1: Caveolin-1
DMEM: Dulbecco’s modified eagle medium
EC: Endothelial cell
ECBM: Endothelial basal medium
ERG: Electroretinography
FITC: Fluorescein isothiocyanate
GCL: Ganglion cell layer
GFAP: Glial fibrillary acidic protein
HUVECs: Human umbilical vein endothelial cells
IBa1: Ionized calcium-binding adapter molecule1
INL: Inner nuclear layer
NG2: Neural/Glial Antigen 2
NVU: Neurovascular unit
OCT: Optical coherence tomography
ONH: Optic nerve head
ONL: Outer nuclear layer
P: Postnatal day
PAS: Period-Acid-Schiff
PBS: Phosphate-buffered saline
PFA: Paraformaldehyde
PLA: Proximity ligation assay
RNFL: Retinal nerve fiber layer
RPE: Retinal pigment epithelium
SLO: Scanning laser ophthalmoscope
TRT: Total retinal thickness
VE-Cadherin: Vascular endothelial cadherin
VEGFR2: Vascular endothelial growth factor receptor type 2
