# Supplementary figures and images for: Sufficient Cav-1 levels in the endothelium are critical for the maintenance of the neurovascular unit in the retina
Source: Mol Med. 2023 Nov 3;29:152. doi: 10.1186/s10020-023-00749-9 (PMC10623831; doi:10.1186/s10020-023-00749-9)

# Supplementary Figure 1

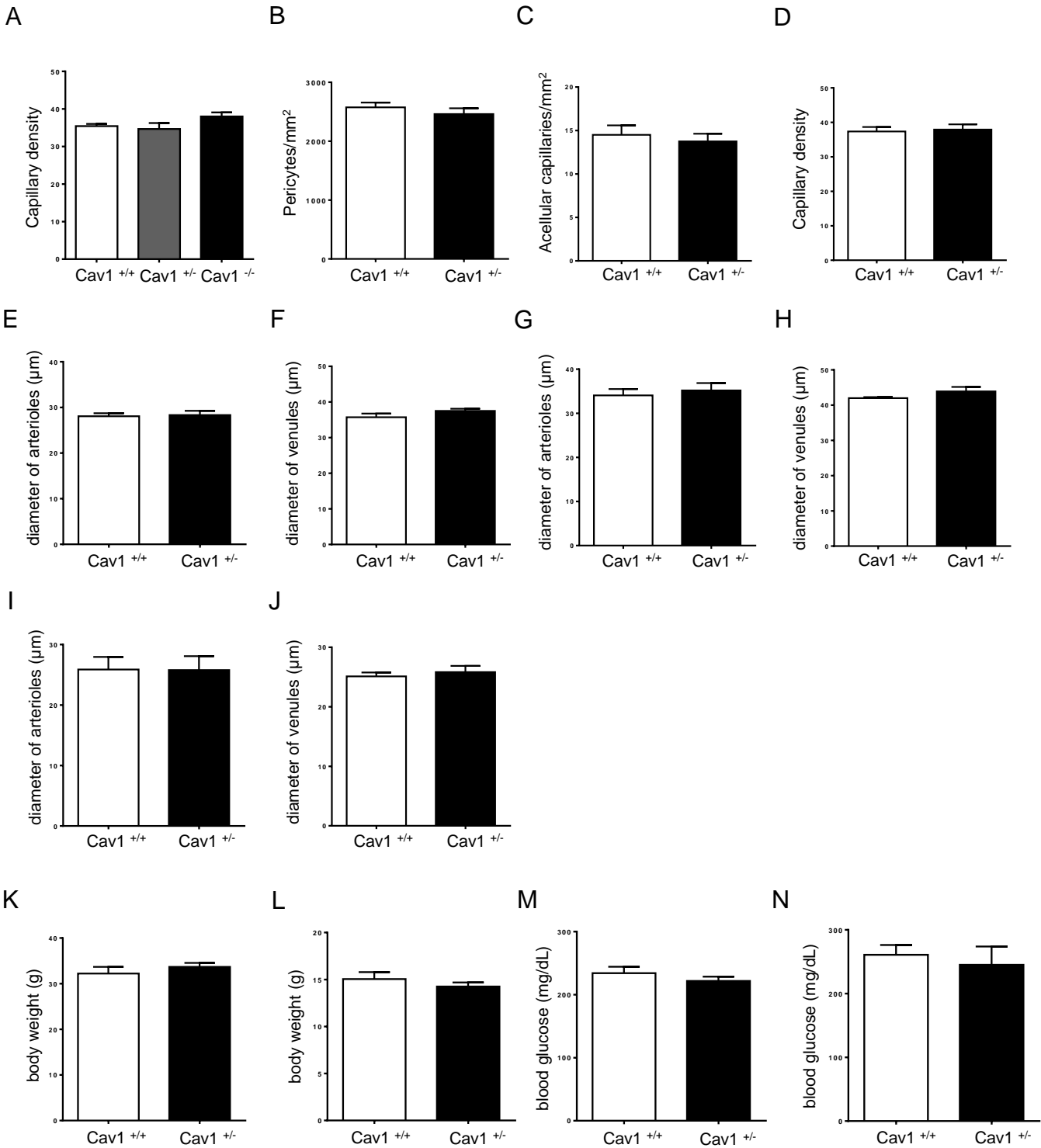

# Supplementary Figure 2

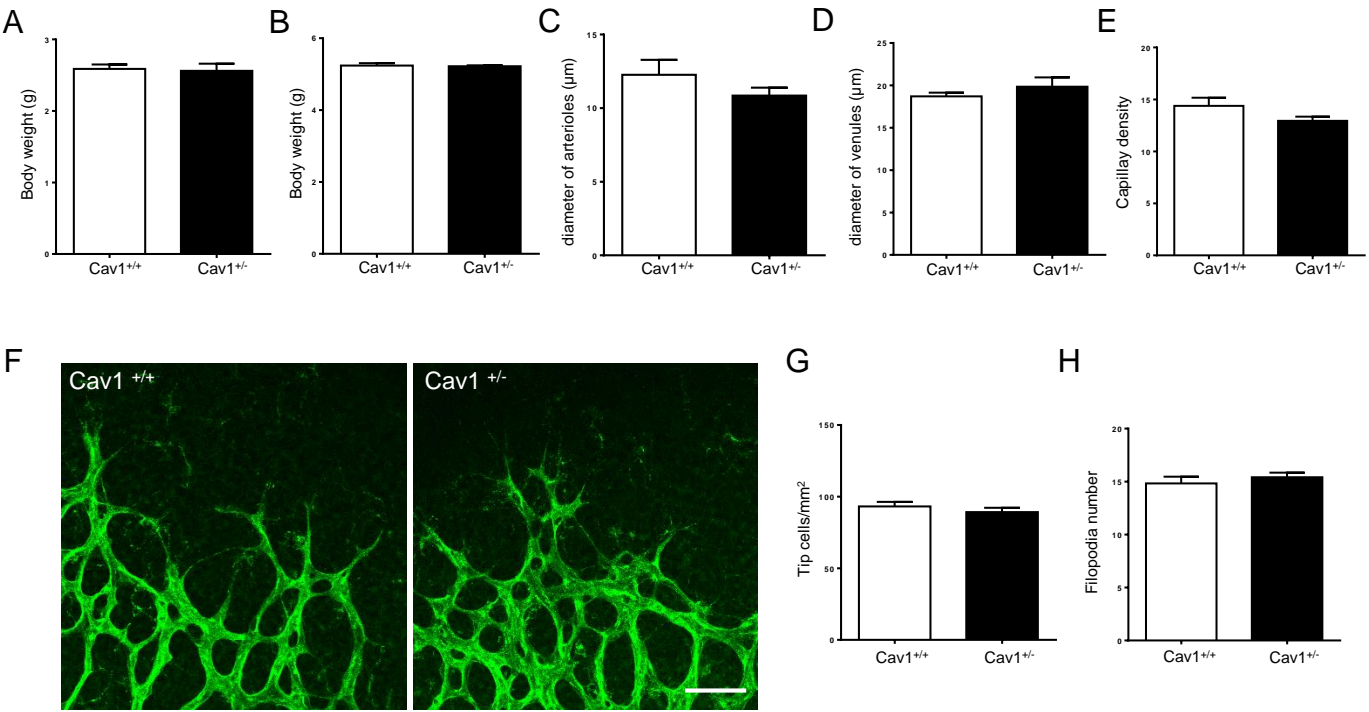

Supplement: Supplementary file 1 — Additional file 1: Figure S1. Vasculature phenotype in 1-month-old retinas, body weight in 1- and 8-month-old Cav-1+/− mice. The quantification of capillary density in 8-month-old Cav-1+/+, Cav-1+/− and Cav-1−/− retinas (A). n = 7–8. The quantification of pericyte coverage (B), acellular capillary number C and capillary density D in 1-month-old Cav-1+/+ and Cav-1+/− retinas. n = 7–8. The quantification of arterioles E and G and venules F and H diameters in 8- E and F and 1-month-old G and H Cav-1+/+ and Cav-1+/− on retinal digest preparation. n = 5. The quantification of arterioles I and venules J diameters in 8-month-old Cav-1+/+ and Cav-1+/− in whole-mount immunofluorescence staining retinas. n = 3. Body weight (K and L) and blood glucose (M and N) of 8- (K and M) and 1-month-old (L and N) Cav-1+/+ and Cav-1+/− mice. n = 8. Figure S2. Body weight and vascular sprouts of Cav-1+/− retinas at p5. Quantification of the body weight of Cav-1+/+ and Cav-1+/− mice at p5 (A) and p10 (B). n = 6. Quantifications of the retinal arteriolar C and venular D diameters and capillary density E at p5 retinas. n = 6. Representative images F and quantifications of retinal vascular tip cells G and filopodia H of Cav-1+/+ and Cav-1+/− retinas at p5. n = 6. Scale bar 50 µm. [file 10020_2023_749_MOESM1_ESM.pdf]
